# Properties and Applications of Geopolymer Composites: A Review Study of Mechanical and Microstructural Properties

**DOI:** 10.3390/ma15228250

**Published:** 2022-11-21

**Authors:** Ahmed Saeed, Hadee Mohammed Najm, Amer Hassan, Mohanad Muayad Sabri Sabri, Shaker Qaidi, Nuha S. Mashaan, Khalid Ansari

**Affiliations:** 1 Department of Civil Engineering, Southeast University, Nanjing 211189, China; 2 Department of Civil Engineering, Zakir Husain Engineering College, Aligarh Muslim University, Aligarh 202002, India; 3 Peter the Great St. Petersburg Polytechnic University, 195251 St. Petersburg, Russia; 4 Department of Civil Engineering, College of Engineering, University of Duhok, Duhok 42001, Iraq; 5 Department of Civil Engineering, College of Engineering, Nawroz University, Duhok 42001, Iraq; 6 Faculty of Science and Engineering, School of Civil and Mechanical Engineering, Curtin University, Bentley, WA 6102, Australia; 7 Department of Civil Engineering, Yashwantrao Chavan College of Engineering, Nagpur 441110, India

**Keywords:** geopolymer composites, clean technology, flexural strength, compressive strength

## Abstract

Portland cement (PC) is considered the most energy-intensive building material and contributes to around 10% of global warming. It exacerbates global warming and climate change, which have a harmful environmental impact. Efforts are being made to produce sustainable and green concrete as an alternative to PC concrete. As a result, developing a more sustainable strategy and eco-friendly materials to replace ordinary concrete has become critical. Many studies on geopolymer concrete, which has equal or even superior durability and strength compared to traditional concrete, have been conducted for this purpose by many researchers. Geopolymer concrete (GPC) has been developed as a possible new construction material for replacing conventional concrete, offering a clean technological choice for long-term growth. Over the last few decades, geopolymer concrete has been investigated as a feasible green construction material that can reduce CO_2_ emissions because it uses industrial wastes as raw materials. GPC has proven effective for structural applications due to its workability and analogical strength compared to standard cement concrete. This review article discusses the engineering properties and microstructure of GPC and shows its merits in construction applications with some guidelines and suggestions recommended for both the academic community and the industrial sector. This literature review also demonstrates that the mechanical properties of GPC are comparable and even sometimes better than those of PC concrete. Moreover, the microstructure of GPC is significantly different from that of PC concrete microstructure and can be affected by many factors.

## 1. Introduction

Concrete is a widely utilised material in the building industry worldwide [1]; because of its low cost, durability, strength, and flexibility to be produced in any shape or size, it is considered the most extensively material used in building [2]. PC is one of the most energy-intensive building materials used in reinforced concrete applications, with current output estimated to be 2.60 billion tonnes (BT) per year worldwide and increasing by 5% annually. PC is made using the “two grinding and one calcining” technology, which uses limestone, clay, and other raw materials as inputs, with a calcination temperature of 1450 °C. Approximately one billion tonnes (BT) of limestone, 180 MT of clay, 50 MT of iron powder, 100 MT of coal, and 60 B KWh of energy are used annually by the cement industry in China [3]. A lot of carbon dioxide is released into the air during cement manufacturing (one tonne of cement production emits approximately one ton of CO_2_). Extremely high carbon dioxide levels are released into the air during the cement manufacturing process (roughly one tonne of cement production releases one tonne of CO_2_). This causes it to account for about 7% of all manufactured CO_2_ emissions. Table 1 [4] summarises the amount of carbon dioxide released during the construction of various concrete elements.

**Figure 1:**
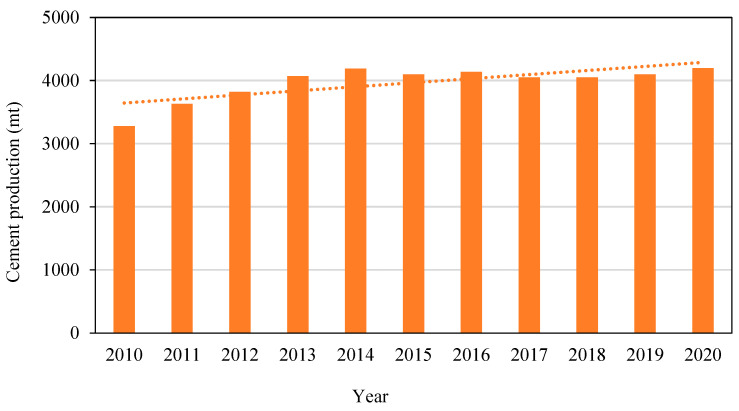
Worldwide cement production in Metric Tonnes, 2010–2020 [7].

Additionally, Figure 1 presents the global trends in growing cement production. Limestone calcining and using a rotary kiln powered by fossil fuels to provide high heat contribute to substantial CO_2_ emissions; this is a significant concern when utilising the PC. One of the most promising options is using geopolymers as a partial or complete substitute for cement [5,6].

**Figure 2:**
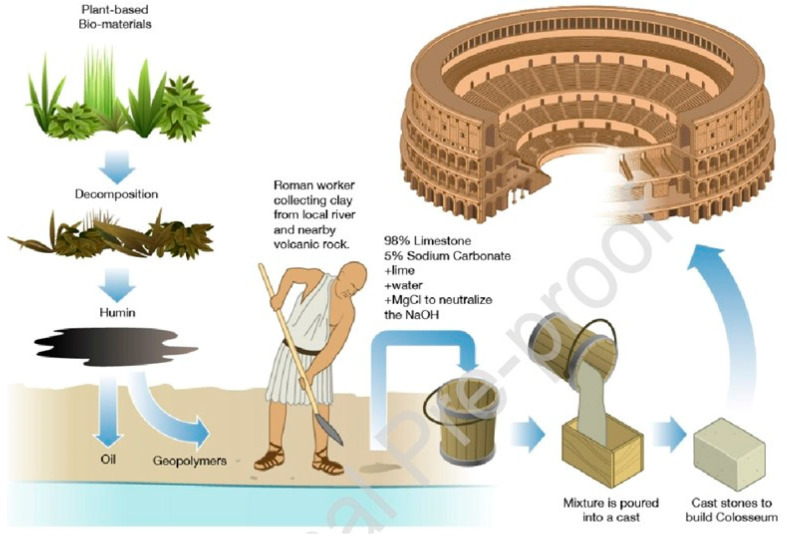
Manufacturing Process of GPCs in the Roman Kingdom [16].

**Figure 3:**
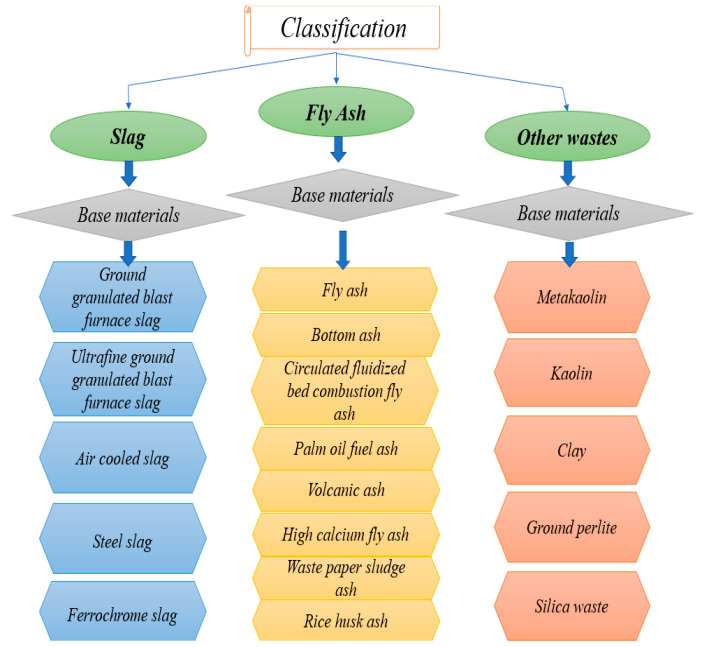
Raw materials used in geopolymer production.

In 1979, Davidovits introduced the term geopolymer to refer to a group of mineral binders similar to zeolites within amorphous microstructure and chemical composition [8]. This material was previously used in the Roman Empire (Figure 2). An aluminosilicate precursor (Figure 3), made up of amorphous silica and alumina, and an activator (a dissolving agent) make up the geopolymers. The term “geopolymerisation” describes the transformation of zeolitic-like materials into a 3D aluminosilicate gel [9,10,11,12]. Generated binder performance is optimised through an alkaline polycondensation process involving silicate and aluminate [13]. It is a common practise to use caustic alkalis of the MOH type or silicates of the R_2_O(n)SiO_2_ type in the synthesis of geopolymers. Sodium hydroxide (NaOH), sodium carbonate (NaCO_3_), potassium hydroxide (KOH), or sodium sulphate (Na_2_SO_4_) are common activators that contain the alkali metal (M) indicated by MOH [14]. In addition to the conventional activators, silica fume and rice husk ash have also been used as activator components in the synthesis of geopolymers [15].

**Figure 4:**
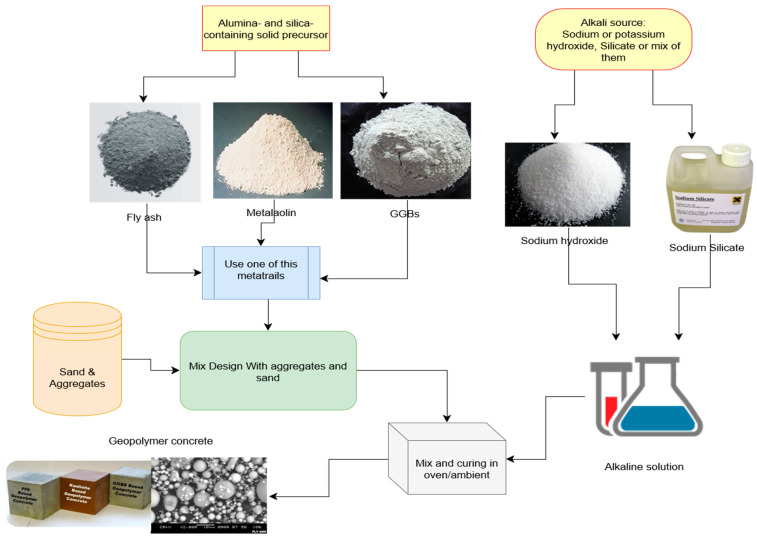
Geopolymer concrete production [25].

The environmental benefits of geopolymer concrete have led many to call it the “next generation” of concrete. It provides a novel approach to reducing CO_2_ emissions in the construction industry by abolishing PC as a binder in concrete production [1,17,18]. Using geopolymer concrete not only has positive effects on the environment and human health but also provides a method for safely disposing of potentially hazardous materials [19]. In addition, research shows that the production of geopolymer concrete can reduce CO_2_ emissions by about 22–72% compared to that of PC concrete at a similar cost [20]. In addition, tests [21,22,23,24] have confirmed that geopolymer concrete has exceptional mechanical properties. Geopolymer concrete (shown in Figure 4) is a sustainable building material depicted in a simplified diagram.

It was observed in the literature that there are many existing review studies to analysing the behaviour of geopolymer concrete [1,2,3,4,5,6,7,8,9,10,11,12,13,14,15,17,26,27,28,29,30,31,32,33,34]. However, previous review publications on geopolymer composite mortar/concrete highlighted mechanical or microstructural properties in the fresh and hardened states. However, they did not provide comprehensive information on this issue in a single paper. In addition, new studies that are relevant to the topic but were not discussed in earlier reviews were published. Therefore, this study provides a comprehensive overview of the most up-to-date studies on geopolymer concrete’s mechanical and microstructural performance. It includes a review, analysis, and discussion of the literature on geopolymer concrete in its fresh and hardened states to aid in the education of researchers and the building industry.

### Study Significance

The published literature papers were evaluated regarding the production of GPC and its mechanical behaviour. This paper discusses the mixed design, mechanical characteristics, and durability of GPC. Using the GPC as an alternative construction material is essential for environmental purposes. Before it can be used in construction, much research needs to be conducted on how structures behave with huge structural elements.

Therefore, this review article aims to provide inclusive information on GPC production, its economic benefits, durability, and environmental influences; the basic process for GPC production; and the factors affecting its mechanical properties.

## 2. Geopolymer Mortar (GPM)

Classic cement mortar is often utilised as a standard binding and repairing material in various engineering structures. Many scholars have addressed GPM’s viability and potential applications as a suitable replacement for regular cement mortar [35]. Sathonsaowaphak was the first to investigate geopolymer mortar and studied the properties of bottom ash fineness, ash/liquid alkali ratio, NaOH/Na_2_SiO_3_ ratio, NaOH dosage, water to ash ratio, and superplasticiser on the behaviour in terms of workability and compressive strength of GPM [36]. Geopolymer mortar has a mechanical strength of 24–58 MPa, and adding NaOH solution improves the workability performance of GPM without reducing strength. According to the results of Detphan and Chindaprasirt [24], who prepared GPC using rice husk ash and fly ash and activated by NaOH and NaSiO_3_ solution as a liquid for the mix, they found that the maximum strength of GPM is acquired by employing a Na_2_SiO_3_-to-NaOH mass ratio of four. Moreover, more discussion about geopolymer mortar properties is reported in the following sections.

### 2.1. Fresh Geopolymer Mortar Properties

#### 2.1.1. Fresh Geopolymer Mortar Workability

**Figure 5:**
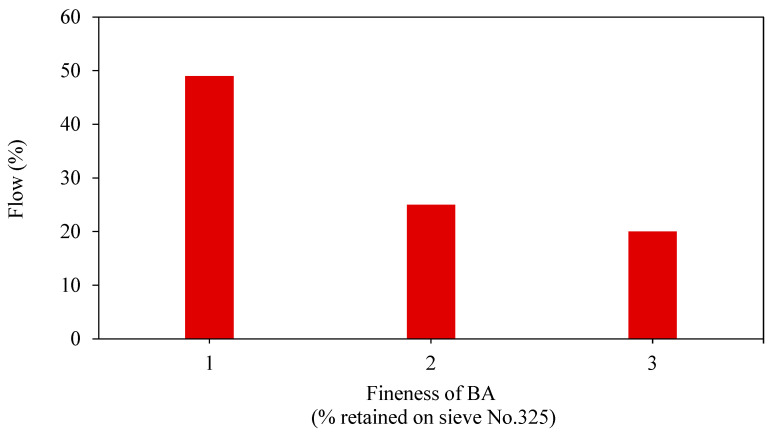
The flow of mortar with various BA fineness [36].

**Figure 6:**
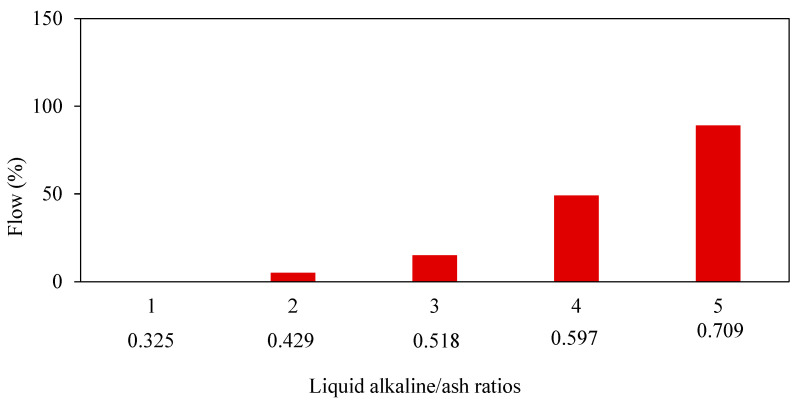
The flow of mortar with various liquid alkaline-to-ash ratios [36].

The workability of fresh GPM is crucial in determining the hardened GPM quality. The concentration ratio of NaOH determines the geopolymer mortar’s workability and the Na_2_SiO_3_ to NaOH. The flowability of modern mortars is typically controlled with the addition of water, which does not compromise the mortar’s strength [37]. Flow, which a flow test may evaluate, is frequently used to determine whether mortar is workable. The term “flow” is widely used to describe how well new mortars work, and it is given as a percentage of the starting base diameter as per the ASTM C1437 standard [38]. Some testing instruments include a flow mould, measuring tape, tamper, flow table, and trowel. The flow test determines a material’s consistency, filling ability, and workability. Sathonsaowaphak [36] studied the effect of bottom ash (BA) fineness on mortar workability and suggested that ground bottom ash might be employed as a raw material for the production of geopolymer. When the fineness of BA was increased, the workability of the mortar was improved. (Figure 5), as well as the impact of various liquid ratios of alkaline/ash. The workability of the mixes improved as the liquid alkaline/ash ratio was raised, as seen in Figure 6.

**Figure 7:**
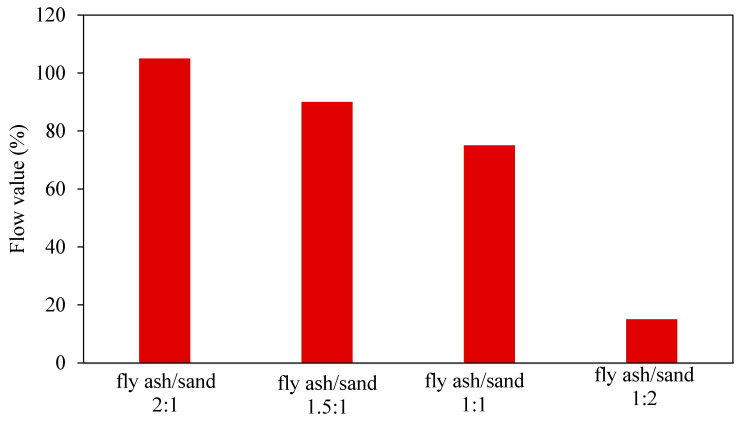
Flow value vs. fly ash/sand ratio [39].

**Figure 8:**
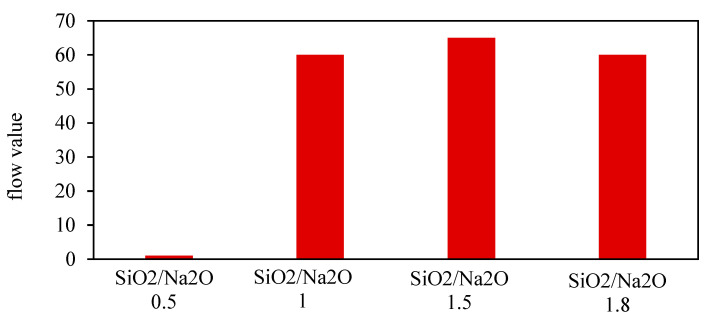
Flow value vs. SiO_2_/Na_2_O ratio in activator [39].

Bhowmick and Ghosh [39] determine the impact of fly ash/sand ratios and the influence of SiO_2_/Na_2_O ratio inactivators on GPM workability. They found that the flow value percentage increases with the fly ash/sand ratio, and the fresh geopolymer mortar’s flowability increases as the SiO_2_/Na_2_O ratio in the activator increases, as shown in Figure 7 and Figure 8.

#### 2.1.2. Fresh Geopolymer Mortar Setting Time

**Figure 9:**
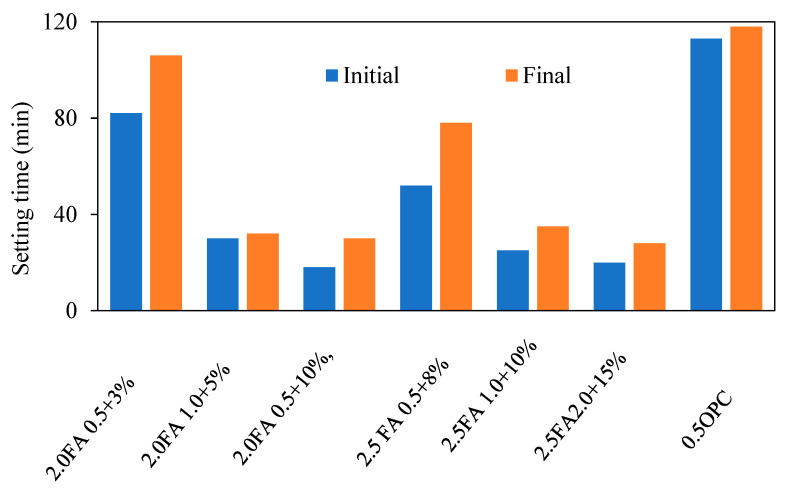
GPM setting times at different mixture proportions [42].

Fresh mortar setting time is critical for transporting, casting, and compacting the mortar within the time restriction. The Vicat needle device can be utilised to test the setting times per the BS EN 480-2 and ASTM C 807-13 standards [40,41]. The first and final setting times are calculated using the needle’s depth as a reference point, which ranges between 2.5 and 4 mm [41,42]. In order to determine the influence of time after mixing on dielectric properties before heat curing, cast specimens were kept in a laboratory at 28 to 29 °C while being protected from moisture loss by a vinyl sheet geopolymer. The dielectric characteristics of GPM were then measured after being mixed for 24 h [42]. Jumrat et al. [42] investigated the setting time of the specimen’s mixture with the addition of water to obtain the standard flow. The results demonstrate that the weight proportions of NS/NH and FA/AS do not affect the initial and final setup periods. The setting time reduces as NS/NH and FA/AS weight ratios increase, as shown in Figure 9 [24]. By increasing the molarity of NaOH, the initial and final setting times of GPM can be greatly decreased [43].

**Figure 10:**
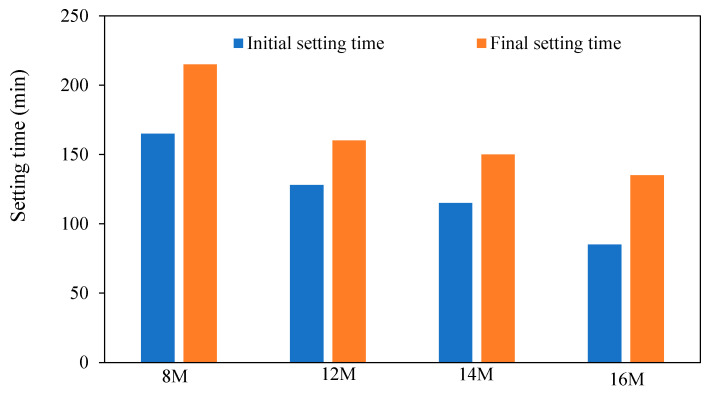
Effect of NaOH molarity on GPM setting times [43].

The setting time reduction in GPM has been attributed to the increased use of PCs. As seen in Figure 10, increased NaOH concentrations caused geopolymer mortars to take longer to set [44].

### 2.2. Geopolymer Mortar Mechanical Properties

#### 2.2.1. Geopolymer Mortar Compressive Strength

Numerous types of source materials are employed as base materials for producing geopolymers. The raw materials used and the proportioning factors impact the strength of GPM. A. Erfanimanesh et al. [45] tried to compare the compressive strength of GPM at the ages of 7 and 28 days, using two different mixed materials (PC mortar, slag, and zeolite). The findings revealed that the compressive strength of GPM increased by up to 48% in the first 7 days compared to the cement mortar after 28 days.

Yusuf et al. [46] studied the effect of blending silica-rich (MK) and palm oil fuel ash (POFA) on the strength of GPM. They indicated that the Weibull distribution is suitable for analysing the blended GPM. Low calcium FA, GGBFS, and POFA can be combined to manufacture GPM under the standard condition that their percentage should be suitable. Ismail et al. [47] investigated the early strength characteristics of a GPM made from palm oil fuel and ash metakaolin with various degrees of NaOH and Na_2_SiO_3_ medium replacement. Ismail et al. [47] studied the compressive strengths of GPM with sisal fibre (SF), coconut fibre (CF), and glass fibre (GF).

Phoo et al. [44], who studied the compressive strength of GPM with different NaOH dosages (6, 10, and 14 mol/dm^3^), found that high calcium FA GPM comprised of PC type I showed increasing PC replacement levels and NaOH concentrations as well as increasing mortar compressive strengths.

A. De Rossi [48] discovered that the strength of geopolymer mortars was affected by the use of construction and demolition waste (CDW) fine aggregates. GPM was formed by combining biomass FA waste and MK as a binder, sodium hydroxide as an activator, alkali sodium silicate solution, and CDW as fine aggregates. Except for the mortar created with particles of 1.0–2.0 mm, when the maximum strength was acquired with sand, CDW was used as aggregate. For CDW–geopolymer mortars, the values were 21 MPa (1.0–2.0 mm), 34 MPa (0.5–1.0 mm), and 40 MPa (0.5–2.0 mm). The mixed fraction had the highest strength values due to the maximum packing density. Table 2 illustrates the effect of various additives on the behaviour of geopolymer mortar.

#### 2.2.2. Geopolymer Mortar Flexural Strength

**Figure 11:**
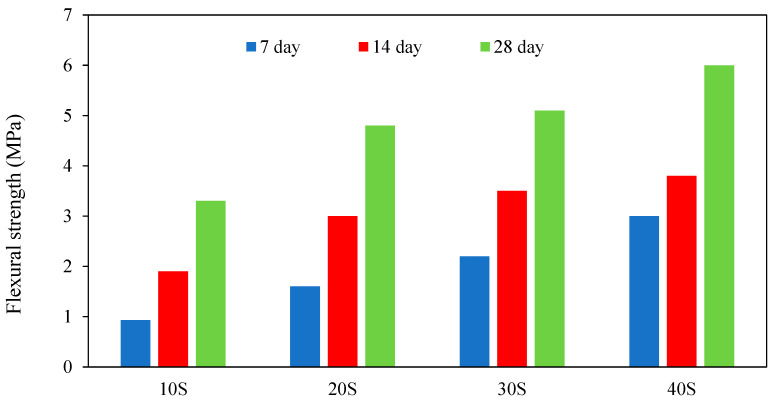
Influence of GGBFS volume on the ultimate flexural strength of GPM [54].

In cement mortars, compressive and flexural strength are tightly linked. However, due to the incredible fragility of the geopolymer and its firm adherence to the aggregate particles, geopolymer mortars have high flexural strength but poor compressive strength [49]. With the addition of sand concentration to 77%, the flexural strength of GPM improves and reaches its maximal value, which slowly decreases because there is an insufficient binder to hold the grains together [20]. Thus, these findings indicate the formation of coarse pores and increased porosity. The alkali activator solution type and curing temperature impact GPM’s flexural strength considerably [50]. According to the results of Huseien et al. [50], the GPM with a curing temperature of 28 °C has higher flexural strength than mortars with curing temperatures of 60 °C and 90 °C. Additionally, the activator solution of sodium aluminosilicate hydrate has lower flexural strength than the sodium hydroxide solution [51]. Li et al. [52] studied the influence of curing conditions on the strength of Class-C FA geopolymer at W/F0.35, where he concluded “For Class-C FA GPM with a water/ash ratio of 0.35 (CF35-C), the findings showed that before the age of 7 d, the non-standard curing shows much higher flexural strength than the standard curing. After steam curing for 24 h and 6 h, flexural strength increased sharply at the age of 1 d; then, strength developed slowly”. Atis et al. [53] studied the flexural strength of GPM with various sodium concentrations and cured it for 24, 48, and 72 h at temperatures ranging from 45 °C to 116 °C. Atis et al. [53] showed the GPM containing 13% sodium after 24 h of heat curing at 116 °C had the maximum flexural strength, while the GPM incorporating 4.0% sodium after 24 h of heat curing at 106°C had the lowest flexural strength. Al-Majidi et al. [54] investigated the effect of Ground granulated blast-furnace slag (GGBFS) content on the ultimate flexural strength of GPM specimens cured at ambient temperature and variations in flexural strength with increasing GGBFS volume at curing ages of 7, 14, and 28 days; the results showed that at all ages, increasing the GGBFS content increased the ultimate flexural strength of GPM significantly. At 7 days, the flexural strength was improved by increasing the GGBFS content from 10 to 20, 30, and 40%, respectively. Flexural strength increased with longer curing durations, with flexural strength values for 10S, 20S, 30S, and 40S combinations rising at 14 and 28 days, respectively, compared to flexural strength values at 7 d. This is seen in Figure 11.

**Figure 12:**
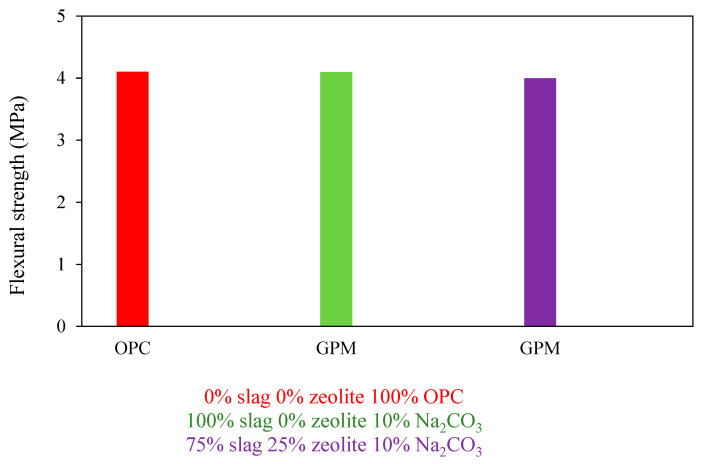
Flexural strength comparison between GPM and PCM.

According to Erfanimanesh et al. [45], flexural strength comparison between GPM and PC mortar (Na_2_CO_3_ concentration was about 10% by weight of powder mixes (zeolite and slag), and 100% Fine Aggregate). GPM’s flexural strength was tested using two distinct mix designs that used slag and zeolite as base ingredients. As shown in Figure 12, the geopolymer and the PC mortars had nearly comparable flexural strengths.

**Figure 13:**
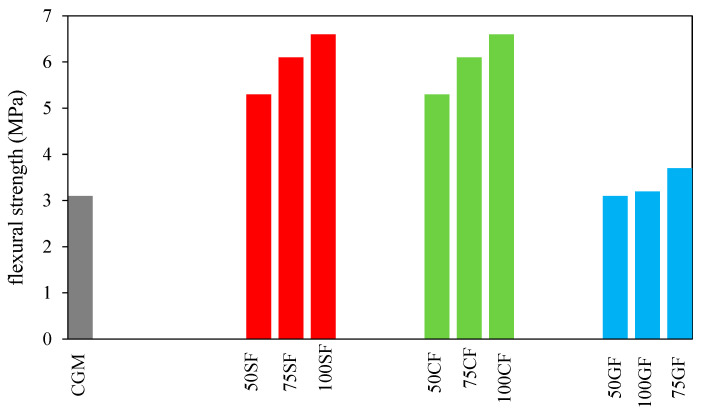
Flexural strength of GPM [55].

Wongsa et al. [55] examined the properties of GPM comprising natural fibres and high levels of calcium fly ash. The primary materials in this investigation included coir or coconut fibre (CF), glass fibre (GF), and sisal fibre (SF). SF and CF were acquired from a plant farm in the Thai provinces of Prachuap Khiri Khan and Chon Buri, respectively. According to the results, utilising fibres enhanced GPM’s flexural strength. In addition, the flexural strength of GPM tended to increase as the fibre content increased. Even though flexural strengths increased with fibre content, the mixtures with more than 1% volume fraction had poor workability and were challenging to compact and cast. The flexural strength of GPM reinforced with natural fibre (CF and SF) varied from 5.3 to 6.6 MPa compared to CGM (3.2 MPa) and GPM reinforced with synthetic fibre (GF) (3.1–3.7 MPa), as shown in Figure 13.

## 3. Geopolymer Concrete (GPC)

GPC is a cost-effective alternative that can be applied in place of standard cement. Thus, using GPC instead of PC cuts CO_2_ emissions dramatically [25]. FA, MK, and GGBFS are examples of GPC sources, including Al and Si as rich materials [56]. GPC consists of three components: GGBFS, or MK, a source of aluminosilicates, such as FA; coarse and fine aggregate; and an activating solution consisting of sodium hydroxide and sodium silicate [20]. GGBS, FA, and MK, among other supplemental cementitious materials, are utilised as binders rather than cement. Using GGBS in combination with PC, FA, and palm oil fuel ash results in a high-strength GPC [16]. FA is the substance most frequently employed in the creation of GPCs as a single source of material [24]. Geopolymer concrete emits 20% less CO_2_ than PC when (5–15%) Portland cement is substituted. This is because geopolymer concrete may attain an early compressive strength of 66 without needing external heat [20]. In order to create geopolymer paste, an inorganic polymeric binder, the source material must be combined with the activator, a solution of NaOH and Na_2_SiO_3_. Despite minimising the cost of GPC, no research on the availability and cost of GPC raw materials has been conducted globally. A global review of raw materials availability has been conducted, focusing on China and the U.S. The demand and cost of each raw material, including FA, SC, MK, NaOH, Na_2_SiO_3_, and silica fume, were analysed for China, the United States, and other major markets for which data are available. This study is essential to see if GPC can be a viable commercial alternative to PC concrete. Raw material supply is a pivotal impediment to widespread GPC adoption [23], so while the cost of each material is listed and briefly addressed, a more in-depth cost study was not conducted.

### 3.1. Fresh Properties of Geopolymer Concrete

#### 3.1.1. Geopolymer Concrete Workability

Umniati et al. [29] highlighted that the workable flow of the GPC increases as the fly ash/sand ratio increases, and the sodium silicate solution’s cohesion and slump ability improve when the SiO_2_/Na_2_O ratio rises [14]. Saranya et al. [23] also used the IS-1199:1959 slump cone test to find out how the different workable samples of GPC were. Due to its dense flow character, the slump value of GPC specimens was 72% higher than that of cement concrete. Steel fibres limit workability because they block the flow of concrete. Mehta et al. [30] studied the workability of GPP, and GPC was investigated at different molarities of NaOH and varied SiO_2_/Al_2_O_3_ ratios by mass. Because the particles on the alkaline activator mixture grow as the dosage of NaOH increases, it is also noted that the dosage of NaOH considerably impacts the workability of GPC.

**Figure 14:**
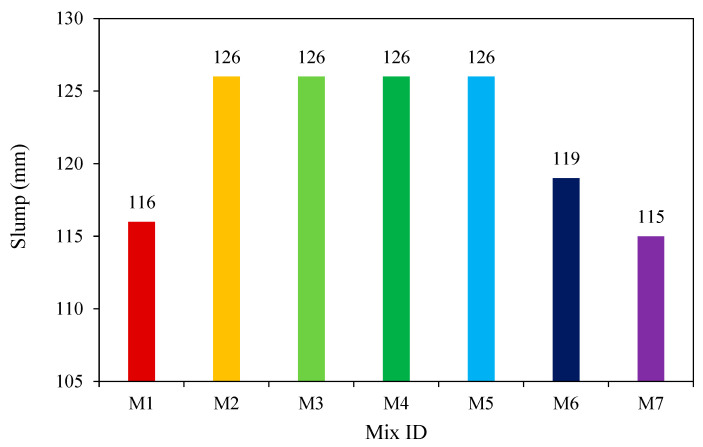
Variation in slump for different mixes (mm) [31].

Hassan et al. [31] evaluated the workability of FA-based GPC using the slump cone test. Coarse and fine aggregate, alkaline liquid, FA, and water are used in GPC. Sodium silicate and sodium hydroxide are combined to make the alkaline liquid. As shown in Figure 14, the findings of the experiments showed that adding GGBS makes GPC less workable.

**Figure 15:**
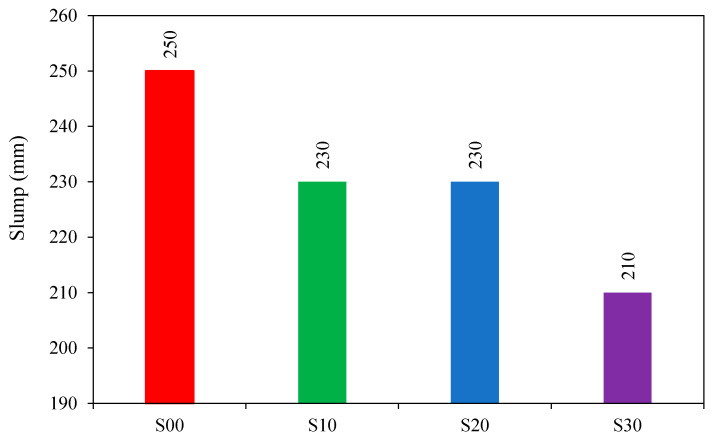
Slump value variation with slag content [16].

Sarker et al. [16] examined the feasibility of fly ash-based GPC for curing in ambient conditions. The experimental results showed that the slump and flow parameters are reduced in FA-based GPC when the slag blend increases. The behaviour becomes more visible as the percentage of the blend increases. As shown in Figure 15, S00, S10, S20, and S30 represent combinations with slag contents of 0%, 10%, 20%, and 30%, respectively.

The steel and iron industries produce ground granulated blast furnace slag (GGBS). According to Patilet et al. [57], using GGBS in concrete increases the material’s workability, among other benefits. Geopolymer concrete made from ground-granulated blast furnace slag was developed by Rajarajeswari et al. [58].

Fumed silica is a byproduct of the silicon and ferrosilicon alloy industries. Khater [59] states that due to its spherical shape, adding fumed silica in concrete increases its density and workability. With the activation of sodium silicate, fumed silica can form dihydrogen during synthesis and can be involved in modifying the chemistry and porosity of specimens.

#### 3.1.2. Geopolymer Concrete Setting Time

**Figure 16:**
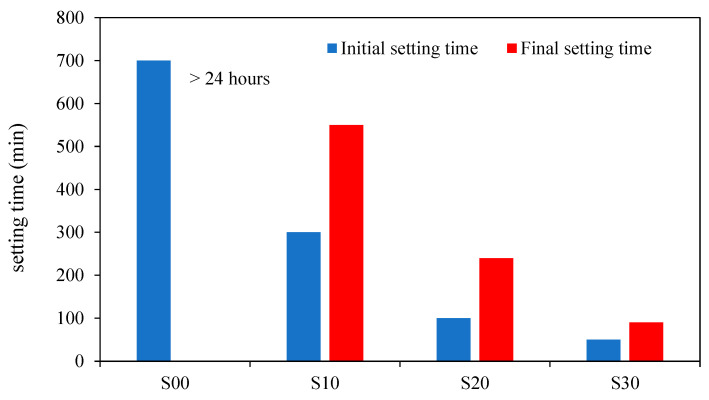
Slag variation during the initial and final setting times [16].

Setting time refers to the time that concrete can be cast, compacted, and transported [14]. As per ASTM C807 and the British Standards Institution (2009), the Vicat needle device determines the concrete setting time. Antoni et al. [32] observed that NaOH concentration influences the curing time of geopolymer concrete. They noticed that reducing the molarity of NaOH could effectively delay the setting of GPC. Because of the slower reaction rate at a low ambient temperature of 20–24 °C, FA-based geopolymer paste takes more than 24 h to set. However, slag blending considerably reduces both the initial and final setting times. When 10% slag is added to the binder, the initial setting time is lowered to 290 min, further reduced to 95 min, and 40 min when the slag concentration is increased to 20 and 30%. The discrepancy between the beginning and final setting times are reduced by increasing the slag concentration in GPP. As shown in Figure 16, the higher the proportion of slag, the faster the setting. With the rise in slag content, Figure 16 depicts the change in initial and final setting times and the difference in the period between the two. S00, S10, S20, and S30 indicate the addition of 0, 10, 20, and 30% GGBFS blends, respectively [16].

Brew et al. [60] produced quick-setting geopolymer concrete with fumed silica at ambient curing conditions. Waste management can be effectively performed by utilising fumed silica in GPC [61]. The addition of 20–30% fumed silica to geopolymer concrete can improve its setting time [62].

### 3.2. Mechanical Properties of Hardened Geopolymer Concrete

#### 3.2.1. Compressive Strength

**Figure 17:**
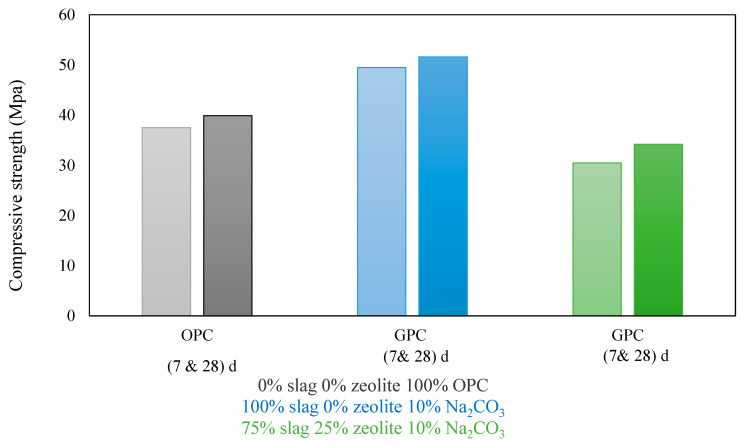
GPC and Portland cement concrete (PCC) compressive strength experiment data (10% Na_2_CO_3_ by weight of powder mixtures (slag and zeolite), 50% fine aggregate) [45].

Compressive strength has usually expressed the behaviour of concrete in compression, and the initial elastic modulus represents the strength development with the shape and age of the stress–strain relationship. Lakshmi and Nagan [33] reported that the wet-mixing time, curing time, curing temperature, and particle size influence geopolymer concrete’s compressive strength (ASTM C 39). Memon et al. [54] found that geopolymer concrete’s compressive strength decreases dramatically when the added water content exceeds 12% of FA mass. After 1 d, heat-cured LCFA-based GPC reaches its maximal compressive strength with no additional increases in compressive strength over time [14]. When cured at 80–90 °C, about 91% of the final strength is formed in just a few hours. Nevertheless, as in PC concrete, GPC cured in an ambient environment setting becomes stronger over time. All curing regimens (ambient temperature) achieve similar long-term strength findings. The amount of time needed to reach the mixture’s maximal compressive strength depends on the curing temperature [14,63]. When employing GPC in the construction of concrete applications subjected to harsh environments, highly high compressive strengths and enhanced durability can be attained [64]. Similar to the relationship between the w/c ratio and the strength of PC concrete, it was found that the ratio of geopolymer particles to water was inversely correlated with the concrete’s compressive strength. The entire mass of geopolymer solids [25] is comprised of binder, sodium hydroxide solids, and sodium silicate solids [27]. Ng et al. [65] provided that the ideal mass ratio for compressive strength in a geopolymer combination of fly ash and slag is 35:65. However, this ratio relies on the reactivity of the particular FA and GGBFS utilised. Bhowmick et al. [49] investigated the impact of FA/sand ratio, SiO_2_/Na_2_O ratio, and water/FA ratio on compressive strength, and according to the findings, the Na_2_SiO_3_/NaOH ratio has an entirely different impact on GPC compressive strength than the SiO_2_/Na_2_O ratio. When the Na_2_SiO_3_/NaOH molar ratio is set at 2.5, there is no significant increase in the compressive strength of GPC. The compressive strength of a structure significantly impacts its stability and safety. The curing conditions and the raw materials impact the compressive strength of FA/GGBS-based geopolymer concrete [66]. Erfanimanesh et al. [45] reported that the zeolite/slag ratio influenced the compressive strength of GPC, as shown in Figure 17. After 7 and 28 days, the GPCs had 30% and 25% higher compressive strengths than the PC concrete. As a result, the mechanical characteristics of the GPC samples were outstanding. Table 3 illustrates the effect of various additives on the behaviour of geopolymer concrete.

#### 3.2.2. Tensile Strength and Flexural Strength

**Figure 18:**
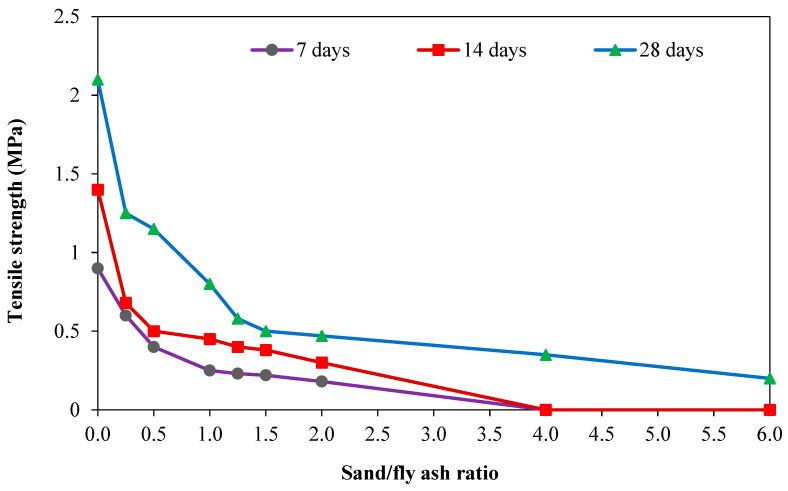
GPC tensile strength of different sand/fly ash ratios [84].

The splitting tensile strength (fsp) and the flexural strength (fr) of GPC increase in tandem with the compressive strength [79], and the compressive strength is proportional to the splitting tensile strength (fsp) and the flexural strength (fr). Test results by Hardijito [63] showed that the splitting tensile strength of geopolymer concrete is only a fraction of the compressive strength. However, there are some deviations from this general response described by some investigators. According to Ryu et al. [14], the rate of tensile strength increase slows as compressive strength increases. Replacing fly ash with GGBS was found to have a lower effect on splitting tensile and flexural strengths as compared with that compressive strength [80]. Tests by Oderji et al. [81] showed a reduction in flexural strength as the fly ash replacement with slag increased from 15% to 20%, knowing that there is a compressive strength enhancement with this modification. Hassan et al. [31] found that in contrast to the elastic modulus of GPC, preheating concrete at 75 °C for 26 h significantly increased compressive and flexural strengths. Other tests by Sarvanan and Elavenil [82] showed that in contrast to the compressive strength if 50% of fly ash is replaced with GGBS, there is a significant splitting tensile strength enhancement. The same observation was made for the elastic modulus property. Comparing data given by Partha et al. [83] with the others showed that using a special heat curing affects enhancing the flexure/compression ratio and, to a lesser degree, the tensile/compression ratio, as compared with the case of ambient temperature curing. Lee et al. [84] presented the tensile strength of the GPC experimental test was measured at 7, 14, and 28 days. Figure 18 demonstrates that when the ratio of sand to FA increases, the tensile strength steadily falls. The outcomes of experimental tests of geopolymer concrete tensile strength were compared by Zhuang et al. [85] based on the design strength of the ACI code, and findings suggest that the splitting tensile strength of GPC is comparable to that of the ACI design. According to Zhuang et al. [85], the tensile strength of the GPC was better than that of PC-quality concrete.

### 3.3. Microstructural Properties of Hardened GPC

#### 3.3.1. X-ray Diffraction (XRD)

X-ray diffraction is a microstructural scanning test performed to investigate and identify a material’s atomic and crystallographic nature. The XRD analysis test involves the irradiation of materials with entrant X-rays and then counting the intensities and scattering angles at which X-rays are emitted; the dispersed rays are expressed in terms of scattering angles, which examines the position of the substance to identify its composition, and identifies scattered intensity peaks. XRD is used in conjunction with other microstructural analyses such as optical light microscopy, electron microprobe microscopy, and scanning electron microscopy in geologic research, mainly if the sample to be analysed is a mixture; XRD data can identify each mineral in a sample and its concentration.

**Figure 19:**
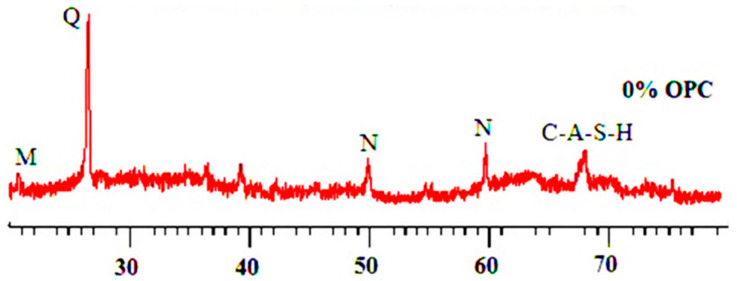
XRD of geopolymer concrete [86].

Different researchers have studied the XRD pattern of geopolymer concrete [66,84,85,86,87,88,89,90,91,92,93,94,95]. Al Bakri et al. [95] noticed that “The basic material of the geopolymer-concrete is of a prevailingly amorphous character only seldom containing needle-shaped minority crystals”. Figure 19 shows the X-ray diffraction (XRD) geopolymer concrete in its as-received condition.

#### 3.3.2. Scanning Electron Micrograph (SEM) and Energy Dispersive X-ray (EDX)

SEM involves examining and scanning the material’s surface with a focused beam of electrons through image analysis to measure and assess fine details. When electrons contact sample atoms, they provide multiple surface topography and composition signals, highlighting component failures, detecting particles, and analysing the interactions between substances and substrates.

**Figure 20:**
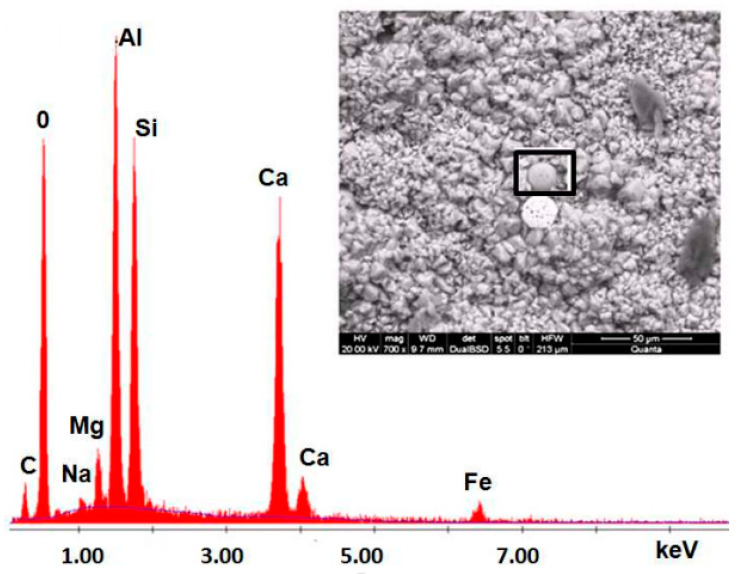
SEM and EDX curve showing the chemical composition of geopolymer concrete [99].

Some studies have investigated the SEM and EDX test of GPC [88,96,97,98,99]. The microstructure of geopolymer-treated concrete appears more refined and denser than untreated concrete (Figure 20). Calcium hydroxide was not detected using EDX, but particles of non-reactant lime were present at levels similar to the control specimen. The main component of geopolymer cement is lime, which explains why it has these properties. Calcium, silicon, magnesium, and potassium peaks increase dramatically in the EDX elemental analysis of the geopolymer-modified PC concrete, which can be attributed to the high concentrations of these elements in the geopolymer cement (except potassium, which originates mostly from the alkaline activator).

**Figure 21:**
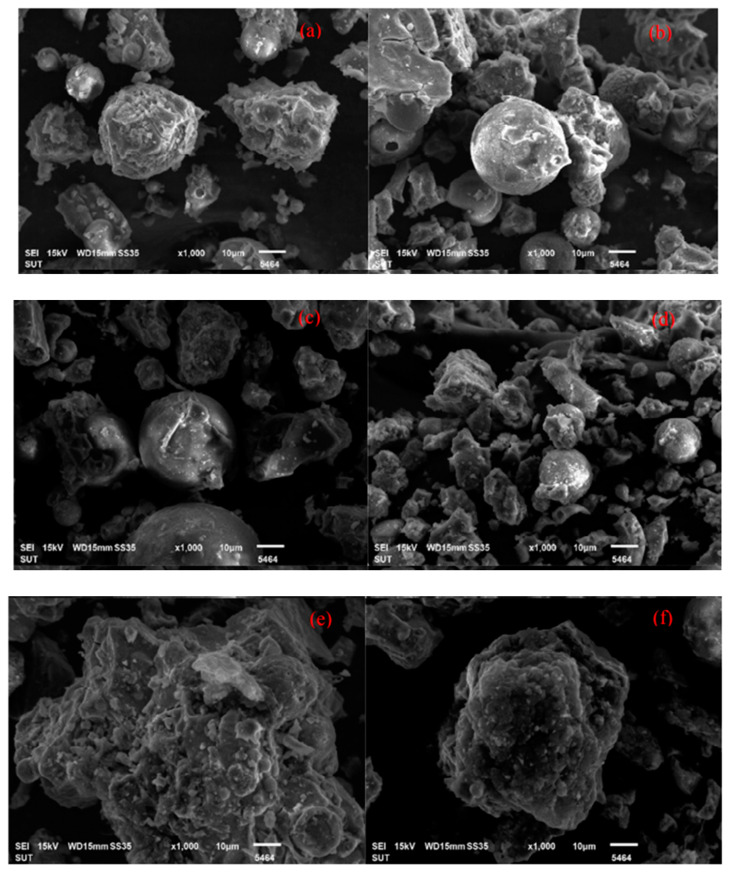
SEM images of a fracture surface of geopolymer nano-composite reinforced with carbon nanotubes (**a**) NaOH 10M control without MWCNTs (**b**) 10M control with CNTs 1 wt% (**c**) NaOH 15M control without MWCNTs (**d**) 15M control with MWCNTs 1 wt% (**e**) NaOH 20Mcontrol without MWCNTs and (**f**) 20M control with MWCNTs 1 wt%. [88].

Jittabut et al. [88] showed that scanning electron microscopy (SEM) pictures of the failed specimens were taken to investigate the geopolymer concrete microstructure. Figure 21 shows the micrographs of geopolymer concrete. The microstructure revealed that several phases exist in the matrix GPC. According to Han et al. [90], the microstructure investigation is absolute, thick, and unbroken, with a highly reactive microstructure. The polymerisation results were in good condition, with no breakdown or crystalline water.

#### 3.3.3. Fourier Transform Infrared Spectroscopy (FTIR)

**Figure 22:**
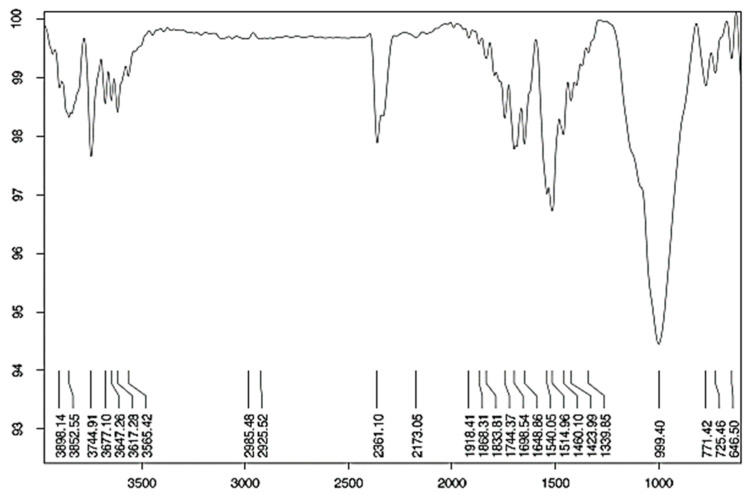
Fourier transform infrared spectroscopy (FTIR) of geopolymer concrete [102].

FTIR techniques are used to measure the infrared absorption, emission, and photoconductivity of a solid, liquid, or gas and identify PHB functional categories. Very limited studies have investigated the FTIR test of GPC [100,101,102]. Figure 22 represents the curing geopolymer sample spectra in the range of 4000 and 400 cm^−1^. Rajini et al. [102] observed that the broad bands in geopolymer mixtures at roughly 3350–3370 cm^−1^ are caused by the tensile vibrations of H-O-H bonds, whereas the broad bands at 1640–1646 cm^−1^ are caused by the bending vibration of the water-associated-OH group.

**Figure 23:**
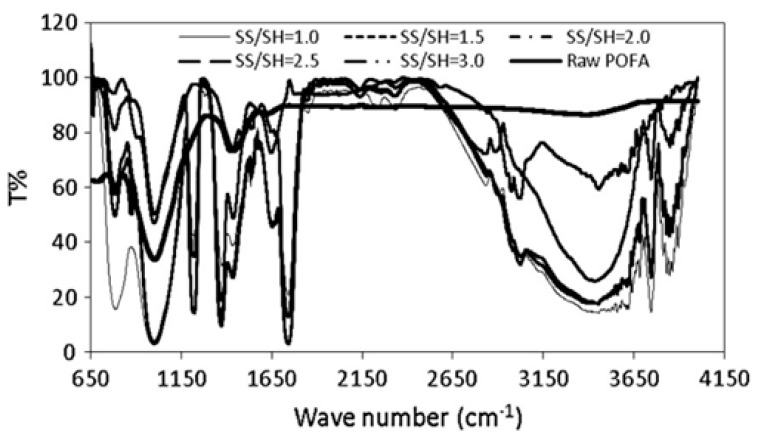
FTIR spectra of raw geopolymerized [101].

Salih, M. A. et al. [101] explain that “the spectra of raw geopolymer paste with different sodium silicate to sodium hydroxide ratios were distinguished with six groups of bands in the regions of 750–850 cm^−1^, 900–1200 cm^−1^, 1200–1300 cm^−1^, 1300–1600 cm^−1^, 1600–1700 cm^−1^, and 2850–3700 cm^−1^ (Figure 23). The first peak was observed at a wave number of 750–850 cm^−1^ centred at 783 cm^−1^, which may refer to the symmetric stretching vibration of Si–O–Si”.

#### 3.3.4. Differential Scanning Calorimetry (DSC)

DSC is an impressive scientific tool for pointing out various physical properties and thermal transitions of polymeric materials. Several distinctive features of the geopolymer pastes can be measured with DSC, which allows observation of exothermic and endothermic processes, in addition to glass transition temperatures (Tg).

**Figure 24:**
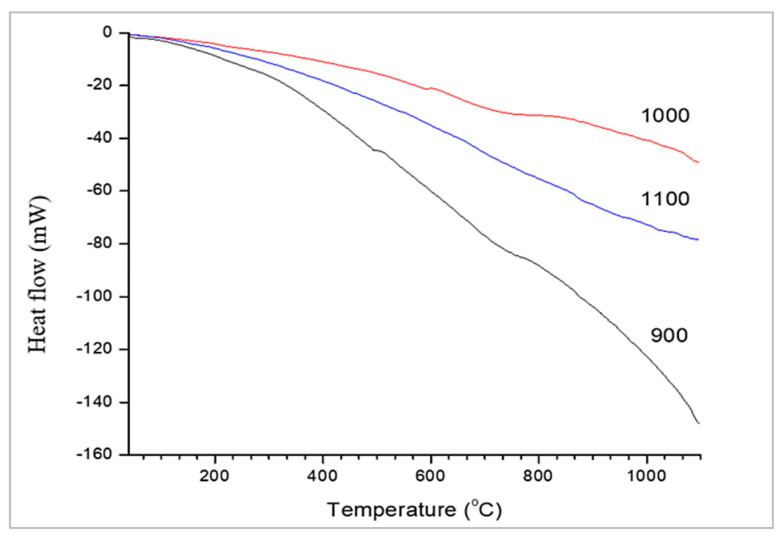
Differential scanning calorimetry (DSC) [107].

Different researchers have studied the DSC analysis of geopolymer concrete [103,104,105,106,107]. Jamil, N. H. et al. [107] showed the geopolymer’s range of thermograms (exothermal up) (Figure 24), where the geopolymer demonstrates some peaks in the matrix.

**Figure 25:**
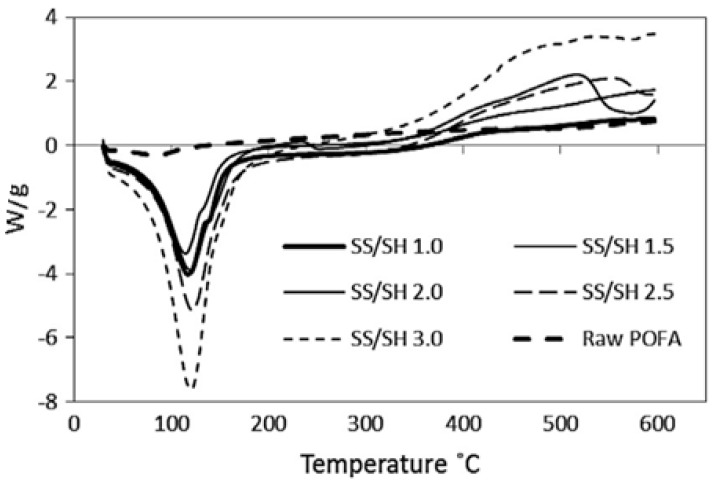
DSC diagrams of raw geopolymerized [101].

Figure 25 depicts the differential scanning calorimetry (DSC) thermograms of geopolymer paste after 28 days, which are similar to the results reported in previous works [101]. From what can be seen in the diagram, there was only one discernible endothermic peak. The pastes with 1:1, 1:1.5, 2:1, 2:1, and 3:1 sodium silicate to sodium hydroxide ratios had this major peak centred at 117.55 C, 114.49 C, 119.40 C, 121.42 C, and 120.02 C, respectively. Water being released from partially dehydrated C-S-H clusters may account for the peaks. For stoichiometric ratios of 1.0, 1.5, 2.0, 2.5, and 3.0 between sodium silicate and sodium hydroxide, the endothermic results were 3888.03, 3,43.106, 3,84.209, 5140.29, and 7410.29 W/g, respectively.

#### 3.3.5. Thermal Gravimetric Analysis (TGA)

TGA aids in the identification of several concrete phases, including calcite, portlandite, C-A-H, C-A-S-H, etc. The hydration reaction is often checked by measuring the CH content; if the CH level decreases, it indicates that CH has been consumed in the hydration reaction. Researchers from several fields have examined the geopolymer concrete TGA [108,109,110,111].

**Figure 26:**
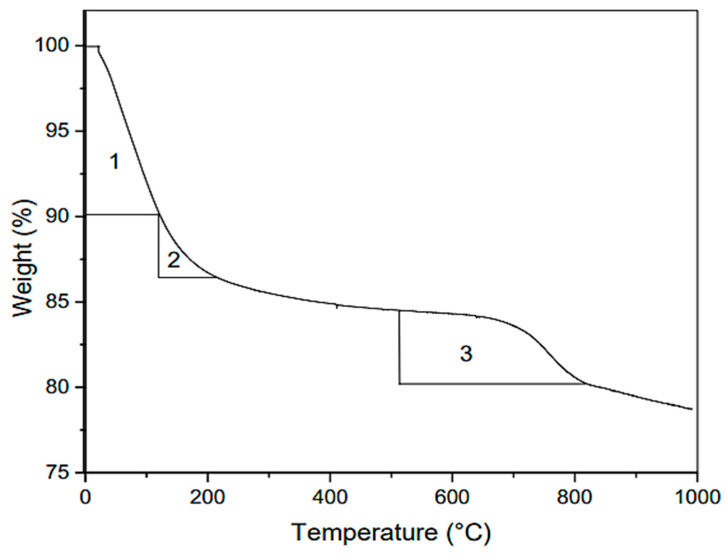
Thermogravimetric (TGA) analysis of geopolymer [94].

Figure 26 shows the TGA analysis of geopolymer concrete’s thermogram. Rosas-Casarez et al. [94] emphasised the primary drop in slope between 0 °C and 1000 °C. In the temperature range of 0 to 120 °C, the dewatering operation resulted in a ten percent weight loss, which is the first substantial drop (number 1 in Figure 26). This process is related to free water that has been adsorbed on the sample’s surface, water that can evaporate from the sample, and the sample’s porosity. However, a current study on the loss of hydrated sodium aluminosilicate gel contradicts this notion. Between 120 and 200 °C, the presence of NASH gel contributes significantly to heat degradation (number 2 in Figure 26). Carbonate loss in mass between 450 and 800 °C may be attributable to carbonates synthesised in the outside environment (number 3 in Figure 26). This method can be used as a comparative method between materials [112,113,114,115,116,117] by relating the water binding capacity for each compound against the effect of temperature. This allows for the differentiation and probing of structural variants and the presence of compounds in a material.

## 4. Scope and Future Research Work

Geopolymer concrete offers much potential in the construction industry. Even though geopolymer concrete has been researched very well in the last few years, several aspects should be covered, and extended data should be collected to understand the behaviour of geopolymer concrete before introducing it to the construction sector. The large-scale structural elements of GPC and the performance of reinforced geopolymer concrete should be investigated. Moreover, the behaviour of geopolymer materials in an aggressive environment, such as a marine environment, must be studied.

Research studies available on structural members of GPC are still lacking. Thus, more investigations need to be carried out on the structural behaviour under the different loading states, and proper modelling is essential, such as developing a proper relationship between the flexural strength, compressive strength, shear strength, and modulus of elasticity of GPC. Moreover, the elasticity and plasticity of geopolymer materials should be well studied to help structural engineers when they are designing buildings. Based on fundamental research, embarking on solid wastes to explore the preparation of varied characteristics of geopolymers in order to produce high-value-added application domains should be one of the primary paths of future geopolymer research development [91,92,111,112,113,114,115,116,117,118,119,120,121,122,123,124,125,126,127,128,129,130,131,132].

## 5. Conclusions

The mechanical properties and microstructural characteristics of geopolymer concrete were reviewed. From the above literature, the following are the final thoughts, along with suggestions for more research:

- Construction with geopolymer concrete is more durable and stronger than with PC concrete;
- Many factors, including curing conditions, the ratio of alkaline to the binder, and the type of activator, have an important impact on the mechanical properties of geopolymer concrete. Consequently, a proper mix design is required to achieve the target strength;
- Geopolymer concrete possesses all the potential characteristics for future applications in civil engineering because it is a green material and requires strength and durability properties for all types of projects in the construction industry;
- Even though it is known that GPC could be used as a replacement material and is a cleaner and more sustainable form of concrete, it is still not widely used in construction;
- Geopolymer concrete needs a standard code to be used more often in building structures;
- In terms of mechanical and microstructural performance, geopolymer concrete was better than PC concrete, especially after exposure to high temperatures;
- The effect of using geopolymer as a partial replacement for PC on the microstructure can be easily noticed; the microstructure has become significantly denser and more homogenous compared with the control specimen, while the number of voids has decreased;
- C-S-H gel and geopolymer gel enhance the mechanical and microstructural properties of precursors that are either high in Ca or contain a combination of Ca components.

## Figures and Tables

**Table 1:** CO_2_ emissions from different concrete volumes [4].

Strength (MPa)	Structural Member	Amount (m^3^)	Emission Factor (tCO_2_^−e^/m^3^)	Emissions (tCO_2_^−e^)
15 32 32 40 40	Blinding Footings Slabs In situ column and wall Precast walls	589 489 1984 253 1067	0.20 0.24 0.27 0.27 0.33	119 119 533 63 351–1185

**Table 2:** Effect of different additives on geopolymer concrete compressive strength.

Ref.	Additives	Remarks
Erfanimanesh et al. [45]	PC mortar, slag, and zeolite	The compressive strength of GPM increased by up to 48% in the first seven days compared to the cement mortar after 28 days.
Tanakorn Phoo [44]	NaOH dosages	A high NaOH dosage increases mortar compressive strengths.
Mohammad Ismail et al. [47]	palm oil fuel and ash metakaolin	The high volume of palm oil fuel and ash metakaolin replacement has been found to reduce compressive strength at an early curing age.
Yusuf et al. [46]	blending silica-rich MK and palm oil fuel ash	The Weibull distribution is suitable for analysing the blended GPM.
Ismail et al. [47]	sisal fibre (SF), coconut fibre (CF), and glass fibre (GF)	The compressive strengths of GPM reinforced with SF, CF and GF both dropped a lot more than those reinforced with SF.
De Rossi [48]	construction and demolition waste (CDW)	The mixed fraction had the highest strength values due to the maximum packing density.

**Table 3:** Effect of different additives on geopolymer concrete compressive strength.

Ref.	Additives	Remarks
Demie, S et al. [67]	Superplasticizer	A high superplasticiser dose increases CS.
Phoo-Ngernkham et al. [68]	Ground granulated BFS	Compressive strength improves when GBFS dosage is increased.
Phoo-Ngernkham et al. [69]	PC mortar	GP composites were developed that have a more uniform and dense structure than concrete.
Islam, A et al. [70]	Ground granulated BFS, palm oil fuel ash	A 67 MPa CS was achieved by combining 30% POFA with 70% GGBS in FA-GP concrete.
Li, Z et al. [71]	Chitosan biopolymer	N-carboxymethyl chitosan’s addition greatly enhanced strength and led to a slight boost in compressive strength.
Yang, T et al. [72]	Ground granulated BFS	The CS of GP mixtures can be increased through the addition of slag to the raw material, with a slag/FA dosage ratio of 0.8, resulting in the highest strength.
Rattanasak, U et al. [73]	Sulfate of calcium and sodium, calcium chloride, and sucrose	The final setting time is significantly prolonged by the presence of sugar. As a rule, admixtures boost CS quality.
Nath, S et al. [74]	GBFS, GCS	Partial replacement with GCS yielded a higher CS than partial replacement with GBFS.
Ding, Y.-C [75]	ground granulated BFS	48 MPa CSs were achieved with an M ratio of 0.96 SiO_2_/Na_2_O and a raw material composition of 70% GGBFS and 30% FA.
Zhang, M et al. [76]	Red mud	There is a decline in CS after 120 days. Safe aggregation of metals where they cannot exceed safe limits.
Kusbiantoro, A et al. [77]	Incinerated rice husk ash	Compressive and bond strength were enhanced when rice husk ash was added at an optimum dosage of 7%.
Torres-Carrasco et al. [78]	Waste glass	Supplemental silicon causes a rise in CS concentration. Typically, 15 g/100 mL is what’s prescribed.

## Data Availability

Not applicable.
